# An objective diagnosis of gout and calcium pyrophosphate deposition disease with machine learning of Raman spectra acquired in a point-of-care setting

**DOI:** 10.1093/rheumatology/keae472

**Published:** 2024-09-02

**Authors:** Tom Niessink, Tim L Jansen, Frank A W Coumans, Tim J M Welting, Matthijs Janssen, Cees Otto

**Affiliations:** Personalized Diagnostics and Therapeutics, Department of Bioengineering Technology, University of Twente, Enschede, The Netherlands; Department of Rheumatology, VieCuri Medical Centre, Venlo, The Netherlands; Department of Rheumatology, VieCuri Medical Centre, Venlo, The Netherlands; Decisive Science, Amsterdam, The Netherlands; Laboratory for Experimental Orthopedics, Department of Orthopaedic Surgery, Maastricht University Medical Centre, Maastricht, The Netherlands; Department of Rheumatology, VieCuri Medical Centre, Venlo, The Netherlands; Personalized Diagnostics and Therapeutics, Department of Bioengineering Technology, University of Twente, Enschede, The Netherlands

**Keywords:** gout, CPPD, diagnostics, synovial fluid analysis, machine learning

## Abstract

**Objective:**

Raman spectroscopy is proposed as a next-generation method for the identification of monosodium urate (MSU) and calcium pyrophosphate (CPP) crystals in synovial fluid. As the interpretation of Raman spectra requires specific expertise, the method is not directly applicable for clinicians. We developed an approach to demonstrate that the identification process can be automated with the use of machine learning techniques. The developed system is tested in a point-of-care-setting at our outpatient rheumatology department.

**Methods:**

We collected synovial fluid samples from 446 patients with various rheumatic diseases from three centres. We analysed all samples with our Raman spectroscope and used 246 samples for training and 200 samples for validation. Trained observers classified every Raman spectrum as MSU, CPP or other. We designed two one-against-all classifiers, one for MSU and one for CPP. These classifiers consisted of a principal component analysis model followed by a support vector machine.

**Results:**

The accuracy for classification of CPP using the 2023 ACR/EULAR CPPD classification criteria was 96.0% (95% CI: 92.3, 98.3), while the accuracy for classification of MSU using the 2015 ACR/EULAR gout classification criteria was 92.5% (95% CI: 87.9, 95.7). Overall, the accuracy for classification of pathological crystals was 88.0% (95% CI: 82.7, 92.2). The model was able to discriminate between pathological crystals, artifacts and other particles such as microplastics.

**Conclusion:**

We here demonstrate that potentially complex Raman spectra from clinical patient samples can be successfully classified by a machine learning approach, resulting in an objective diagnosis independent of the opinion of the medical examiner.

Rheumatology key messages

- The gold standard in synovial fluid analysis, polarized light microscopy, is subjective and unreliable.
- Our machine learning algorithm allows rheumatologists to easily interpret sophisticated Raman spectroscopic analyses.
- The specificity of Raman spectroscopy combined with objective interpretation leads to more certain diagnoses.

## Introduction

In rheumatology, the presence of crystals and particles in joint (synovial) spaces can lead to fulminant inflammation through stimulation of the innate immune system [1, 2]. Monosodium urate (MSU), which is causative of gout, and calcium pyrophosphate (CPP), which is causative of CPP deposition disease (CPPD), are the most common pathological crystals in synovial fluid of patients with these conditions. Polarized light microscopic) identification of the respective crystals in synovial fluid is sufficient to diagnose gout or CPPD in a swollen, painful or tender joint [3–7].

In polarized light microscopy (PLM), a trained rheumatologist identifies negatively birefringent needles (MSU) or positively birefringent rods and rhomboids (CPP) [3, 8, 9]. However, this method requires extensive training, is subjective and is sensitive to artifacts, all of which contribute to low inter-rater reliability [10–13]. Especially, CPP crystals are notoriously difficult to identify [10]. Mismanaged gout increases the risk of cardiovascular disease [14, 15] and overall mortality [14, 15], and can lead to permanent joint damage [16]. Alternative tools for synovial fluid analysis are in development [17], and of these Raman spectroscopy is one of the most promising [18–25]. Raman spectroscopy is a highly specific form of crystal analysis, which is based on inelastic scattering of monochromatic laser light [22]. The atoms in a molecular bond make small oscillatory motions, and in Raman spectroscopy, there is an interaction between laser light and these vibrational bands. As the energy associated with these vibrations is unique per bond, the colour shift resulting from the interaction is a direct representation of the components and structure of the molecule. The interpretation of Raman spectra is not standard practice for rheumatologists and is therefore a barrier for clinical implementation. Although Raman spectroscopy is more objective than PLM due to the unique crystal spectra, methods that require human classification of Raman spectra still might be biased.

Raman spectra are known to be highly suitable for machine learning approaches. Raman spectra have a high-dimensional feature space, which is ideal for machine learning algorithms to extract patterns from. Furthermore, the high specificity of Raman spectra allows algorithms to differentiate between subtle chemical differences. Possible approaches range from basic classification algorithms such as K-nearest neighbour, random forests and linear discriminant analysis to more sophisticated methods such as 1D convolutional networks [26–28]. For pathological crystals, support vector machines (SVM) applied to Raman spectra can already accurately classify microcalcifications in breast cancer [29]. SVM models are well suited for use in Raman spectroscopy and are often combined with either conjoint (specified) or disjoint (unspecified) dimensional reduction [30]. Raman spectra from crystals in biological samples often suffer from high background signals and poor signal-to-noise ratio. Sequences to pre-process Raman spectra from biological samples for machine learning have been established, and have been found to reduce variation between patients [31].

Here we demonstrate a combined approach of Raman spectroscopy and machine learning to provide objective and reliable diagnoses of gout and calcium pyrophosphate-associated arthritis. Of the in total 446 patient samples from three clinical centres, 246 samples were used as a training database for our algorithm. The resulting algorithm was validated with the remaining 200 patient samples. All these samples were measured in a point-of-care setting.

## Methods

### Study design and participants

For creation and testing of the synovial fluid crystal database, 446 patient samples were collected from three centres. Eligible for inclusion were patients above the age of 18 with any swollen peripheral joint who underwent arthrocentesis as part of routine clinical care. Four hundred samples were collected fresh from the outpatient department of VieCuri Medical Centre (VC; Venlo, the Netherlands) between April 2022 and November 2023. Forty-six samples were collected frozen, of which 10 were from the biobank of the Department of Orthopedics of University Medical Centre Groningen (UMCG; Groningen, the Netherlands) and 36 from the Department of Orthopedic Surgery of Maastricht University Medical Centre (MUMC; Maastricht, the Netherlands). All fresh samples were analysed as soon as possible after fluid draw (mean 1.1 day, s.d. 1.6) and stored at room temperature in the syringe. The frozen samples from MUCM and UMCG were stored at −80°C for up to several months before analysis.

Only clinical waste material was used for analysis and patients gave written informed consent before collection of their body material and clinical data. The study was performed according to good clinical practice guidelines and the Declaration of Helsinki. VC samples were considered clinical waste material, and the collection was therefore exempted from ethical review by the METC MUMC+ ethics board. The ethics board of MUMC+ approved the collection in Maastricht (METC permit 2017–0183) and the ethics board of UMCG approved the collection in Groningen (METC permit 2021/407).

### Raman spectroscopic analysis

For each patient sample, a droplet of synovial fluid (10–40 µl) was placed on a microscope slide (Epredia, Kalamazoo, MI, USA) and covered with a coverslip (Menzel-Gläser, Epredia, Kalamazoo, MI, USA). Every sample was then analysed with an integrated Raman polarized light microscope (iRPolM) Raman spectroscope (Hybriscan Technologies B.V., Nijkerk, the Netherlands). The device was located at the outpatient rheumatology clinic of VieCuri Medical Centre. With this device, birefringent crystals and particles were located manually (×20 magnification, 0.85 NA objective) and then individually scanned with the integrated Raman spectroscope. Depending on crystal size, 60–120 pixels were measured with an integration time of 1 s per pixel. The device operates a laser with a 532 nm excitation wavelength and the measured spectrum has a spectral bandwidth of 0–3600 cm^−1^. Measurements were performed with a laser power of 11.3 mW (as measured under the objective). For the creation of Raman intensity images, we chose Raman bands which are specific for MSU (630 cm^−1^) and CPP (1050 cm^−1^), although the classification was performed on the full Raman spectrum. The minimal used pixel size in the Raman spectroscope was 1 × 1 µm.

### Training and validation sets

The samples were consecutively measured over a 2-year time interval. The training set consisted of the frozen samples from UMCG (*n* = 10), MUMC (*n* = 36) and a 50% split of the fresh set from VC (*n* = 200), totalling 246 samples. The validation set consisted of the other 50% of fresh samples from VC (*n* = 200) and therefore was analysed prospectively. The split was made based on the order of presentation at the clinic (consecution), and the final half of the samples collected at VC were used as a validation set. All measurements in the training set were manually analysed for spectral quality such that measurement errors and possible anomalies could be excluded. Spectra with device artifacts (for example the LED of the microscope), high fluorescence backgrounds (for example when samples were burnt) and poor focus were considered outliers and were removed from the training set. The resulting spectra in the training set were assigned three possible categories: MSU, CPP or negative.

### Spectral preprocessing

All collected spectra underwent the same preprocessing algorithm, which was programmed in MATLAB R2023b (MathWorks, Natick, MA, USA). First, cosmic rays were removed. Then, calibration was applied according to device standards: charge coupled device (CCD) camera offset, CCD camera dark current, background, and National Institute of Science and Technology (NIST) intensity calibration. The wavenumber calibration was then applied against the Neon and polymethylmethacrylate (PMMA) reference spectra, and spectra were then aligned to fit a predefined wavenumber axis with linear interpolation (MATLAB function *interp1*) and then shifted to fit the zero-wavenumber laser response. A baseline correction was then applied with a Whittaker smoother algorithm [32]. Spectra were truncated to the interval of 50–3000 cm^−1^ and normalized by dividing the spectrum with its integral. The collected hyperspectral images contained both foreground (information) pixels and background pixels. For hyperspectral images with crystals in the training set, only the foreground pixels can be used for the database. To separate background pixels from those containing crystal information, a 2-cluster *k*-means cluster analysis was performed. The cluster with the highest mean variance was the cluster with crystal information and was added to the database. If the difference in variance of the clusters was <10%, both results from the *k*-means cluster analysis were foreground. For the validation set, all measured spectra were included (no cluster analysis), and no manual preselection was applied. An example of the spectral processing is given in Supplementary Fig. 1, available at *Rheumatology* online.

### Algorithm design

We designed two one-against-all classifiers in Python (3.10.9), one for MSU and one for CPPD. One-against-all classifiers are known to be more accurate than multi-class classifiers [30]. Furthermore, a combination of MSU and CPP present in the same Raman spectrum is possible in mixed crystal disease [33]. Using only data related to MSU or CPP, a principal component analysis (PCA) dimensional reduction model was fitted (Scikit-Learn 1.3.0, [34]). This model was then applied to the complete training set reducing the spectra to only contain information on the 10 PCA dimensions most relevant for either MSU or CPP (disjoint dimensional reduction). Based on this reduced data set, a support vector machine (SVM, Scikit-Learn 1.3.0, radial basis function kernel [34]) model was fitted to classify MSU from other, or CPP from other. Similarly, the validation dataset was dimensionally reduced with the previously trained disjoint PCA models. With the trained SVM models, spectra were predicted to the predefined classes MSU or other, and CPP or other. A patient was considered positive for MSU or CPP if one or more spectra are positively classified. We analysed the training set with t-stochastic neighbour embedding (t-SNE) (Scikit-Learn 1.3.0 [34]) to identify trends and clusters in the data. We set the perplexity to 30, early exaggeration to 12 and performed 5000 iterations.

### Analysis

To assess the comparability between the validation set and the training set, patient characteristics were compared with Fisher’s exact test and unpaired two-tailed Student’s *t*-test. We considered the two sets comparable on a given characteristic if the *P*-value was <0.05.

For both gout and CPPD, identification of crystals in the synovial fluid (both extracellular and intracellular) with PLM is sufficient for a clinical diagnosis. We therefore first compared the outcome of our classification model with the outcome of PLM analysis by a trained rheumatologist. The rheumatologist had to be certain of their analysis to be able to score the sample as crystal positive. In a second analysis, we also regarded the ACR/EULAR classification criteria. The clinical data for determination of the ACR/EULAR score were retrieved after Raman spectroscopic analysis from the patients’ electronic health records. If patients are negative for MSU in PLM but have over 8 points on the 2015 ACR/EULAR gout classification criteria [5], they were considered gout positive. If patients are negative for CPP in PLM but have over 56 points on the 2023 ACR/EULAR CPPD classification criteria [4], they were considered CPPD positive. Both criteria sets use additional clinical data such as lab values, age and radiology. Diagnostic accuracy measures (sensitivity/recall, specificity, positive predictive value/precision, negative predictive value, positive likelihood ratio, negative likelihood ratio, accuracy) with corresponding 95% confidence intervals were calculated using 2 × 2 contingency tables. We calculated the diagnostic accuracy for gout, CPPD and their combination (corresponding results in both tests). We compared results between the different scenarios with two-tailed *Z*-tests for comparing proportions and considered *P*-values below <0.05 as statistically significant.

## Results

Of the 446 samples which were analysed with Raman spectroscopy, the results of 246 (55.1%) samples were included in the training set and the results of 200 (44.9%) samples were included in the validation set. The 246 (55.1%) samples in the training set included 67 gout, 30 CPPD and 149 other arthritis types. The 200 (44.9%) samples in the validation set included 71 gout, 30 CPPD, one mixed crystal arthritis (both MSU and CPP crystals in the synovial fluid) and 98 other arthritis types. There were significantly more patients with osteoarthritis in the training set compared with the validation set (*P* = 0.03), and there were significantly more punctured MTP1 joints in the validation set (*P* = 0.01). While CPPD samples were nicely distributed over training and validation sets, the number of gout samples was higher in the validation set, although this was not statistically significant (*P* = 0.06) (Table 1).

**Table 1.** Patient characteristics of the validation set and the training set

	Total	Training set	Validation set	*P*-value
Patient count	446	246	200	
Clinical diagnosis, *n* (%)^a^				
Gout	138 (30.9)	67 (27.2)	71 (35.5)	0.06
CPPD^b^	50 (11.2)	30 (12.2)	20 (10.0)	0.45
Mixed crystal disease^c^	1 (0.2)	0 (0.0)	1 (0.5)	0.99
Rheumatoid arthritis	40 (9.0)	25 (10.1)	15 (7.5)	0.41
Osteoarthritis	71 (15.9)	47 (19.1)	24 (12.0)	**0.03**
Bacterial infection	11 (2.5)	4 (1.6)	7 (3.5)	0.24
Unknown	135 (30.3)	77 (31.3)	62 (31.0)	0.99
Punctured joint, *n* (%)				
MTP1^c^	77 (17.3)	32 (13.0)	45 (22.5)	**0.01**
Knee	223 (50.0)	131 (53.3)	92 (46.0)	0.15
Wrist	23 (5.2)	16 (6.5)	7 (3.5	0.20
Ankle	43 (9.6)	24 (9.8)	19 (9.5)	0.99
Other	80 (17.9)	43 (17.5)	37 (18.5)	0.81
Age, mean (s.d.), years	66.2 (15.2)	65.7 (15.1)	66.7 (15.4)	0.49
Sex, *n* (%)				0.24
Male	272 (60.9)	144 (58.5)	128 (64.0)	
Female	174 (39.1)	102 (41.5)	72 (36.0)	

a Primary indication.

b Calcium pyrophosphate associated arthritis.

c First metatarsophalangeal joint. *P*-values below 0.05 (shown in bold) were considered significant. CPPD: calcium pyrophosphate deposition disease.

A total of 15 186 Raman spectra were measured in the 246 samples of the training set. The number excluded was 5924 spectra, and these include spectra of low quality and non-crystal spectra in hyperspectral images containing crystals. In total, the training database contained 9237 spectra, 1447 of which (15.6%) were classified as MSU and 1165 of which (12.6%) were classified as CPP. Other clinically important crystals such as calcium oxalate and basic calcium phosphate (BCP) were less prevalent in the sample series and therefore also less frequent in the training database (<5%). A frequent contribution to the database consisted of Raman spectra of microplastics (24.7%), which are frequently present in synovial fluid samples but are at this point of unclear clinical relevance [35]. Calcium carbonate crystals and titanium dioxide particles are a novel finding with Raman spectroscopy and are frequently present in synovial fluid samples [20, 21].

**Figure 1.**
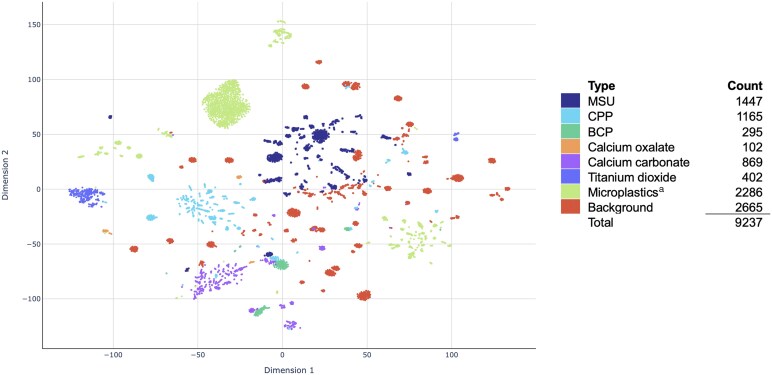
t-Distributed stochastic neighbour embedding (t-SNE) plot visualizing the content of the training set. In t-SNE, data are grouped according to a stochastic dimensional reduction. Dimensional reduction and plot created with Scikit-Learn Python library. Count refers to the number of Raman spectra of a specific type in the database. ^a^The classification microplastics includes polyethylene, polyethylene terephthalate, polypropylene and polystyrene. BCP: basic calcium phosphate; CPP: calcium pyrophosphate; MSU: monosodium urate

The t-SNE plot in Fig. 1 demonstrates how the spectra are distributed over rudimentary groups. In the t-SNE plot, we can see very distinct clusters of background. These mostly represent data from individual measurements containing several spectra. The background group represents not only artifacts but also Raman spectra from, for example, erythrocytes, leukocytes and cartilage filaments present in the synovial fluid. We can see tight clusters of the microplastics, and these have very distinct Raman spectra. An important point to notice is a major overlap between MSU and the background spectra, which indicates potential difficulties in discrimination. If erythrocytes are present in the synovial fluid, they might produce a Raman background signal that partly overlaps with MSU (Supplementary Fig. 2, available at *Rheumatology* online). Furthermore, while BCP, CPP and calcium carbonate crystals have similar Raman spectra, and in the t-SNE plot they mostly cluster in distinct groups.

In the 200 consecutive samples of the validation set, 11 797 Raman spectra were included. Of the 200 samples, 61 were positive for MSU according to polarized light microscopy, and an additional 11 samples could be classified as retrieved from gout patients according to the 2015 ACR/EULAR gout classification criteria [5]. Nineteen samples were positive for CPP according to PLM, and an additional two samples could be classified as retrieved from CPPD patients according to the 2023 ACR/EULAR CPPD classification criteria [4]. In the validation set, one patient was positive for both criteria sets.

For MSU, the classification model had a precision (positive predictive value) of 93.2% (95% CI: 79.8, 96.3), a recall (sensitivity) of 90.2% (95% CI: 83.9, 97.3) and an accuracy of 95.0% (95% CI: 91.0, 97.6) with respect to PLM (Table 2). If we also consider patients with >8 ACR/EULAR gout classification points, the model had a precision of 96.6% (95% CI: 87.8, 99.1), a recall of 81.4% (95% CI: 70.3, 89.7) and an accuracy of 92.5% (95% CI: 87.9, 95.7) (Table 3). Not all patients with gout have MSU crystals in their fluid and the recall is therefore lower when also regarding clinical symptoms. There were two patients negative for PLM that were classified positive by both the model and the classification criteria.

**Table 2.** Diagnostic performance of the combined approach of Raman spectroscopy and automated spectral classification for gout and CPPD with respect to polarized light microscopic analysis of the synovial fluid by an experienced rheumatologist

	MSU, as classified	CPP, as classified	Both, as classified	None, as classified
MSU, as per PLM	52	1	2	5
CPP, as per PLM	0	18	0	0
Double positive, as per PLM	1	0	0	0
Double negative, as per PLM	4	4	0	113

Against PLM	Gout	CPPD	Combined
Sensitivity/recall (95% CI), %	90.2 (79.8, 96.3)	100 (81.4, 100)	88.6 (79.5, 94.7)
Specificity (95% CI), %	97.1 (92.8, 99.2)	96.2 (92.2, 98.4)	93.4 (87.4, 97.1)
Positive likelihood ratio (95% CI)	31.3 (11.9, 82.6)	26.0 (12.6, 53.8)	13.4 (6.8, 26.3)
Negative likelihood ratio (95% CI)	0.10 (0.05, 0.22)	—	0.12 (0.07, 0.23)
Positive predictive value (95% CI), %	93.2 (83.9, 97.3)	72.0 (55.4, 84.2)	89.7 (81.7, 94.5)
Negative predictive value (95% CI), %	95.7 (91.3, 98.0)	100 (97.9, 100)	92.6 (87.1, 95.9)
Accuracy (95% CI), %	95.0 (91.0, 97.6)	96.5 (92.9, 98.6)	91.5 (86.7, 95.0)

Patients are scored as positive for MSU or CPP when one or more spectra measured from their synovial fluid sample is identified as MSU. CPP: calcium pyrophosphate; CPPD: calcium pyrophosphate deposition disease; MSU: monosodium urate; PLM: polarized light microscopy.

**Table 3.** Diagnostic performance of the combined approach of Raman spectroscopy and automated spectral classification for gout and CPPD with respect to internationally accepted disease classification criteria sets

	MSU, as classified	CPP, as classified	Both, as classified	None, as classified
Gout, 2015 ACR/EULAR criteria	54	1	2	14
CPPD, 2023 ACR/EULAR criteria	0	19	0	1
Double positive, both ACR/EULAR criteria	1	0	0	0
Double negative, neither ACR/EULAR criteria	2	3	0	103

Against the ACR/EULAR classification criteria	Gout	CPPD	Combined
Sensitivity/recall (95% CI), %	81.4 (70.3, 89.7)	90.5 (69.6, 98.8)	81.1 (71.5, 88.6)
Specificity (95% CI), %	98.4 (94.5, 99.8)	96.7 (92.8, 98.8)	93.6 (87.3, 97.4)
Positive likelihood ratio (95% CI)	52.9 (13.3, 210.3)	27.0 (12.1, 60.0)	12.8 (6.2, 26.3)
Negative likelihood ratio (95% CI)	0.19 (0.12, 0.31)	0.10 (0.03, 0.37)	0.20 (0.13, 0.31)
Positive predictive value (95% CI), %	96.6 (87.8, 99.1)	76.0 (58.8, 87.6)	91.3 (83.5, 95.6)
Negative predictive value (95% CI), %	90.8 (85.8, 94.2)	98.9 (95.9, 99.6)	85.8 (79.8, 90.3)
Accuracy (95% CI), %	92.5 (87.9, 95.7)	96.0 (92.3, 98.3)	88.0 (82.7, 92.2)

Patients are scored as positive for MSU or CPP when one or more spectra measured from their synovial fluid sample is identified as MSU. CPP: calcium pyrophosphate; CPPD: calcium pyrophosphate deposition disease; MSU: monosodium urate.

For CPP, the classification model had a precision of 75.0% (95% CI: 57.6, 86.9), a recall of 94.7% (95% CI: 74.0, 99.9) and an accuracy of 96.5% (95% CI: 92.9, 98.6) with respect to PLM (Table 2). If we also consider patients with >56 ACR/EULAR CPPD classification points, the model had a precision of 76.0% (95% CI: 58.8, 87.6), a recall of 90.5% (95% CI: 69.6, 98.8) and an accuracy of 96.0% (95% CI: 92.3, 98.3) (Table 3). As with MSU, the recall is lower when also regarding clinical symptoms. The overall precision of the model for CPP was significantly lower than that of MSU (*P* = 0.0036).

If we only consider samples in which both the classifications (CPP and MSU) match with PLM as correctly classified, the classification model had a precision of 86.6% (95% CI: 78.3, 91.8), a recall of 92.1% (95% CI: 83.6, 97.1) and an accuracy of 91.5% (95% CI: 86.7, 95.0) with respect to PLM (Table 2). If we also consider the ACR/EULAR classification criteria with the same criterion (both correct), the model had a precision of 91.3% (95% CI: 83.5, 95.6), a recall of 81.1% (95% CI: 71.5, 88.6) and an accuracy of 88.0% (95% CI: 82.7, 92.2) (Table 3).

**Figure 2.**
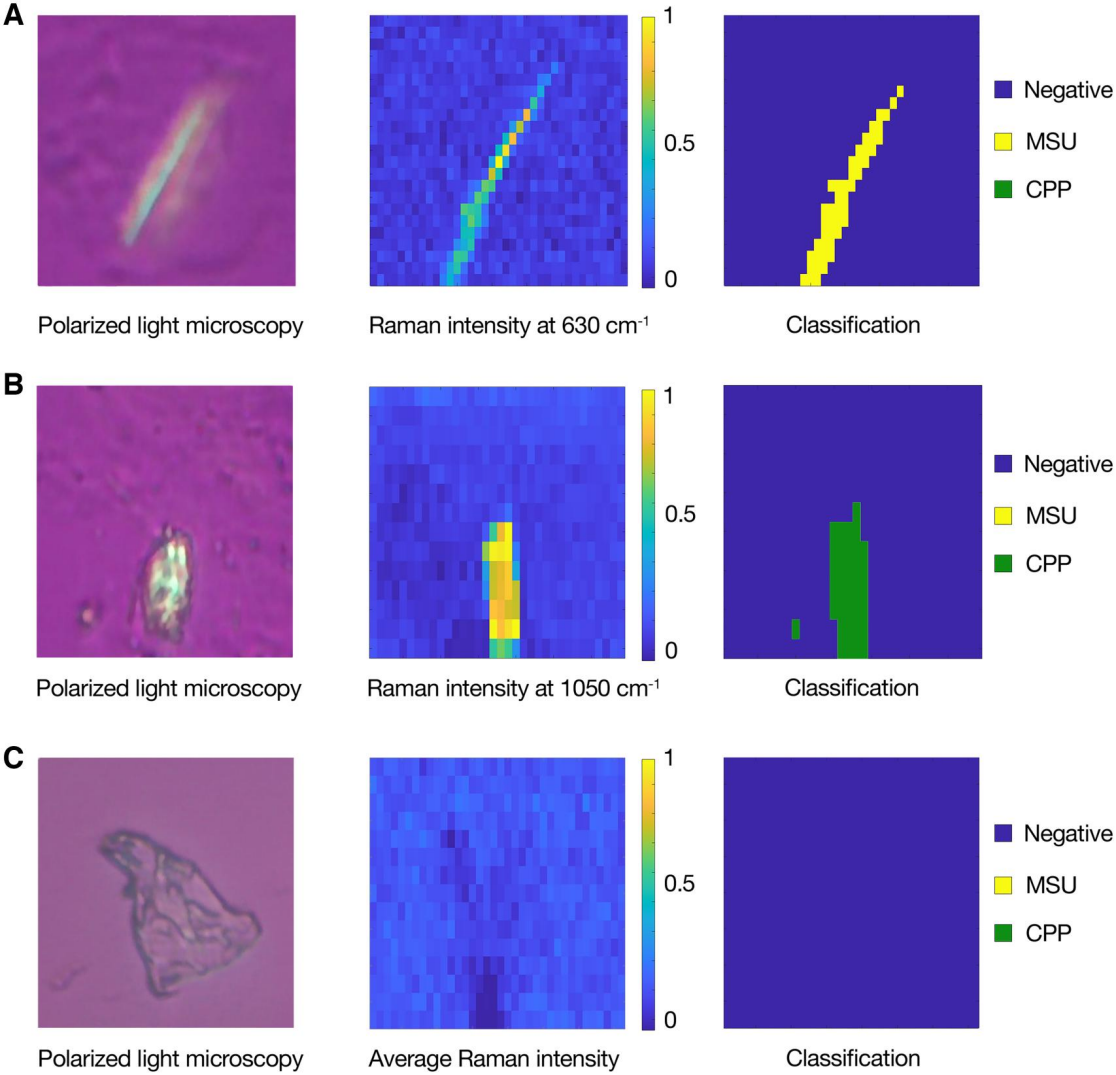
Classification of hyperspectral Raman images measured from crystals in synovial fluid samples. Per image, 400–800 pixels were measured, with a total measurement time of 10–30 min. (**A**) Polarized light microscopic image, Raman intensity heatmap and classification image of a MSU needle. (**B**) Polarized light microscopic image, Raman intensity heatmap and classification image of CPP. (**C**) Polarized light microscopic image, Raman intensity heatmap and classification image of a glass artefacts. Polarized light microscopic images were shifted to fit the Raman intensity heatmap. Microscopic images were made with a Zeiss Axiolab 5 polarized light microscope; Raman spectra were measured on a iRPolM Raman spectroscope (Hybriscan Technologies, Nijkerk, the Netherlands). CPP: calcium pyrophosphate; MSU: monosodium urate

The algorithm performs pixel-wise classification, which therefore also can be used in imaging. In Fig. 2 it can be seen that the classification discriminates crystal pixels from background pixels in high-resolution hyperspectral images. The morphology of the crystals is conserved in the Raman spectral heatmap as the classification image. The combined approach of Raman spectroscopy and algorithmic crystal classification is even able to identify intracellular MSU needles and tiny (<2 µm^2^ based on the Raman resolution) CPP crystals. The overall processing time of a sample with Raman spectroscopy was 15 min. The algorithmic classification can be applied instantaneously and does not increase sample processing time. All Raman spectroscopic data and corresponding classifications in this study are used for research purposes only. We have no adverse events to report.

## Discussion

We have demonstrated that both gout and CPPD can be objectively diagnosed with a machine learning algorithm acting on a large set of >20 000 Raman spectra from synovial fluid samples of 446 patients. The designed models reached an accuracy of 92.5% for gout and 96.0% for CPPD, which is comparable to the diagnostic accuracy of Raman spectroscopy when spectra are classified by a trained observer [20, 22]. The availability of a reliable and objective diagnostic measure for crystal-associated arthritis can be a practice-changing method for rheumatologists worldwide, as the method can be applied in a point-of-care setting.

An important strength of our present study is the large population of 446 included patients. The training database contained a wide variety of patients from three centres including both native joints and prosthetic joints. The trained algorithm was then tested on a consecutive and unselected series of 200 new patients that were not seen earlier by the model, which overall minimizes bias. Based on our statistical testing, the training set and the validation set were an unbiased distribution from the patient pool. We also tested the algorithm against internationally accepted disease classification criteria sets for gout and CPPD, such that the widest acceptable range of clinical factors is regarded in the classification of disease.

The algorithm performed with high accuracy and was able to positively diagnose three patients that were missed by the respective ACR/EULAR disease classification criteria sets. There are a few examples of misdiagnoses with our combined approach of Raman spectroscopy and machine learning. Not all patients with gout and CPPD were positive for crystals in their synovial fluid, which is a partial explanation for the false-negative outcomes of the classification model [4, 5]. In both cases, the recall was higher when only regarding the outcome of PLM with the model compared with the complete criteria set including symptoms. False positives were more common in CPP than MSU. The Raman spectrum of CPP contains about six specific peaks unique to CPP while the spectrum of MSU contains over 20. As with any classification model, a small chance of error remains and clinicians should always regard all available data when determining their clinical diagnosis.

While the model can accurately classify MSU and CPP, these are not the only pathological crystals of interest in synovial fluid. Especially BCP crystals, which are strongly associated with osteoarthritis [36, 37], are of high interest as these cannot be identified with conventional microscopic methods [38]. Whilst some BCP crystals were encountered and analysed within the training cohort, their prevalence was too low to train a BCP crystal classification model. A more focused collection of synovial fluid samples is required to increase the availability of BCP spectra in the training and validation databases.

As a limitation of this study, it should be remarked that only a single classification method was selected while a large variety of (Raman) spectral classification models are available [39]. Machine learning approaches are in rapid development [27, 28, 31], and the number of alternative approaches to the PCA-SVM we applied here keeps growing. However, the dataset is insufficiently large to allow comparison of multiple approaches, as this will lead to problems with generalizability. For training of more advanced methods, larger datasets are required. The reason we selected PCA-SVM for this study is that it is known to be robust in comparable applications [30]. PCA-SVM is known to perform well in cases with a clear margin of separation between classes, such as with Raman spectra. It can also handle high-dimensional data well and is memory efficient. Furthermore, SVM is reasonably interpretable, which may enable the improvement of the detection hardware. Hence our decision to demonstrate only one approach and not compare a multitude of approaches. In this work we validated our model with samples from one clinical centre, which might limit the generalizability of our results. Additional studies with independent samples from different hospitals and regions (external validation) will be required for FDA/IVDR certification of our technology. Eventually, the certified method can be implemented for clinical use.

Overall, the combination of Raman spectroscopy and algorithmic spectral classification provides a strong diagnostic performance for both gout and CPPD. Raman spectroscopy is insensitive to artifacts due to increased chemical specificity. With the addition of algorithmic classification, the method becomes fully objective and no longer requires specific training in the interpretation of the analytical results. The implementation of Raman spectroscopy combined with algorithmic classification in clinical practice may allow rheumatologists to treat gout and CPPD with high certainty of a correct diagnosis.

## Supplementary Material

keae472_Supplementary_Data

## Data Availability

The data that support the findings of this study are available from the corresponding author, T.N., upon reasonable request.
