# Supplementary material for: An objective diagnosis of gout and calcium pyrophosphate deposition disease with machine learning of Raman spectra acquired in a point-of-care setting
Source: Rheumatology (Oxford). 2024 Sep 2;64(4):1791–8. doi: 10.1093/rheumatology/keae472 (PMC11962871; doi:10.1093/rheumatology/keae472)
Supplement: keae472_Supplementary_Data [file keae472_supplementary_data.docx]

**Supplementary figure 1**


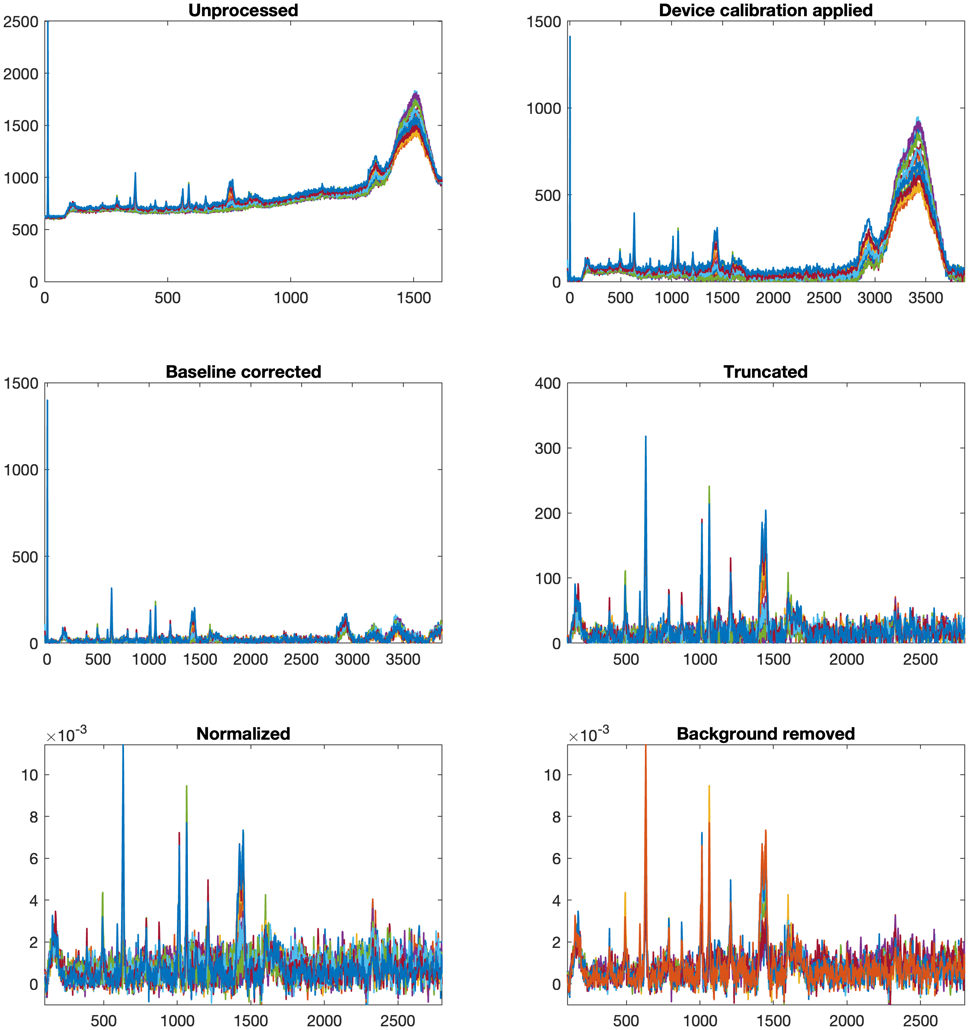


***Supp. Figure 1: Overview of the spectral processing process.*** *Given is a measurement of a MSU crystal which was added to the database. Unprocessed spectra are first calibrated with the daily calibration measurements performed with the device, which removes most of the baseline signal and adds the wavenumber calibration. Then, a Whittaker smoother algorithm corrects for any deviations in the baseline. The spectrum is then truncated, normalized, and using a k-means cluster analysis, any present background is removed.*

**Supplementary figure 2**

**
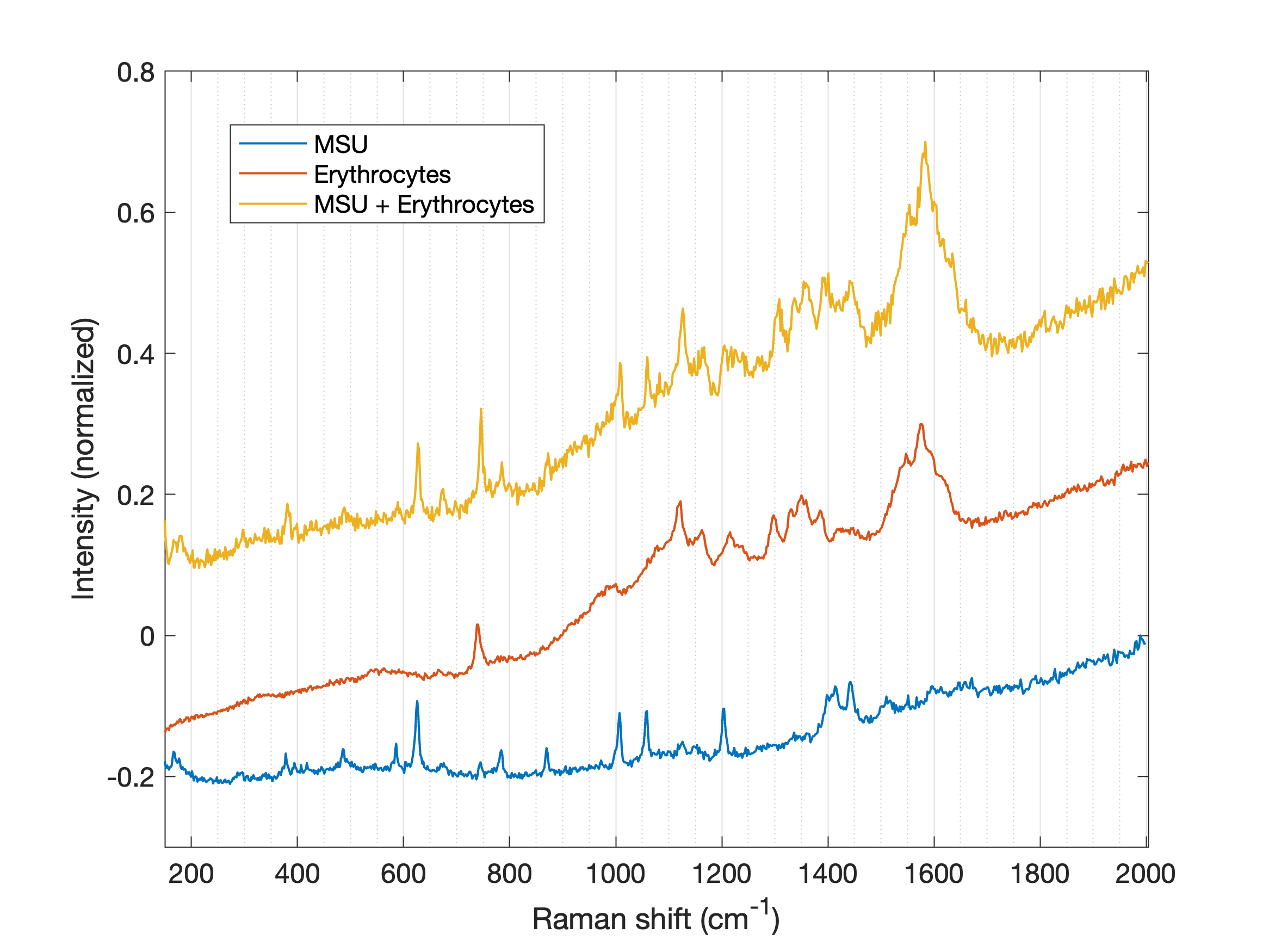
**

***Supp. Figure 2: Raman spectra of monosodium urate (MSU), erythrocytes, and a field of view with both MSU crystals and erythrocytes.*** *All Raman spectra are measured with the iRPolM Raman spectroscope (Hybriscan Technologies), given are normalized spectra without any preprocessing. Visible is how the two spectra partly overlap in the Raman fingerprint region (1000-1500 cm^-1^). This makes visual classification of the spectra more difficult, as the algorithm needs to be able to work around these properties.*
